# Identification of Novel QTL Governing Root Architectural Traits in an Interspecific Soybean Population

**DOI:** 10.1371/journal.pone.0120490

**Published:** 2015-03-10

**Authors:** Lakshmi P. Manavalan, Silvas J. Prince, Theresa A. Musket, Julian Chaky, Rupesh Deshmukh, Tri D. Vuong, Li Song, Perry B. Cregan, James C. Nelson, J. Grover Shannon, James E. Specht, Henry T. Nguyen

**Affiliations:** 1 Division of Plant Sciences, University of Missouri, Columbia, Missouri, United States of America; 2 Department of Agronomy and Horticulture, University of Nebraska, Lincoln, Nebraska, United States of America; 3 Soybean Genomics and Improvement Lab, USDA-ARS, Beltsville, Maryland, United States of America; 4 Department of Plant Pathology, Kansas State University, Manhattan, Kansas, United States of America; Huazhong university of Science and Technology, CHINA

## Abstract

Cultivated soybean (*Glycine max* L.) cv. Dunbar (PI 552538) and wild *G*. *soja* (PI 326582A) exhibited significant differences in root architecture and root-related traits. In this study, phenotypic variability for root traits among 251 BC_2_F_5_ backcross inbred lines (BILs) developed from the cross Dunbar/PI 326582A were identified. The root systems of the parents and BILs were evaluated in controlled environmental conditions using a cone system at seedling stage. The *G*. *max* parent Dunbar contributed phenotypically favorable alleles at a major quantitative trait locus on chromosome 8 (Satt315-I locus) that governed root traits (tap root length and lateral root number) and shoot length. This QTL accounted for >10% of the phenotypic variation of both tap root and shoot length. This QTL region was found to control various shoot- and root-related traits across soybean genetic backgrounds. Within the confidence interval of this region, eleven transcription factors (TFs) were identified. Based on RNA sequencing and Affymetrix expression data, key TFs including MYB, AP2-EREBP and bZIP TFs were identified in this QTL interval with high expression in roots and nodules. The backcross inbred lines with different parental allelic combination showed different expression pattern for six transcription factors selected based on their expression pattern in root tissues. It appears that the marker interval Satt315–I locus on chromosome 8 contain an essential QTL contributing to early root and shoot growth in soybean.

## Introduction

Soybean is a major crop that plays an important role in food and industrial production [1]. The USA ranks first in soybean production [84.2 million metric tons], accounting for 33% of the total global production, followed by Brazil at 29% and Argentina at 19% (www.soystats.com). Soybean production is affected considerably by water deficits and severe drought conditions [2]. Root size and architecture are important factors for determining yield performance, particularly under conditions of limited water availability [3]. Drought resistance in plants is achieved by three different mechanisms: drought escape, avoidance, and tolerance [4]. Plants that utilize the avoidance mechanism endure drought by balancing turgor through increased rooting depth, better root architecture, and increased hydraulic conductance [5]. The intrinsic ability of the plant roots to extract water from deeper soil profiles enables plants to maintain optimal water relations, as well as carbon assimilation, under drought stress [6]. Deep tap roots, with greater density of lateral roots that increase the total root absorption surface area, contribute to drought avoidance in rice [7, 8], maize [9], wheat [10], common bean [11], chickpea [12], and soybean [13, 14, 15].

Studying root architecture and identifying genes underlying its function is critical to develop soybean that suited to water-limited environments [16]. However, several practical constraints associated with root phenotyping under field conditions make it an uncommon practice in soybean breeding [17, 18]. Molecular markers have been widely used to identify quantitative trait loci (QTLs) for complex agronomic traits [19]. Mapping QTLs for root traits and their use in marker-assisted breeding (MAB) (for example, moving a favorable QTL allele present in exotic germplasm into elite cultivars) is an alternative method for selecting root traits that are difficult to phenotype [20]. Rapid screenings of root traits at the seedling stage facilitate identification of contrasting lines to map root QTLs in soybean [21]. Then molecular breeding programs can be targeted to incorporate alleles that produce desired root phenotypes into elite cultivars to ensure productivity under stress environments. Unfortunately, the genetic base of modern soybean cultivars in North America is narrow, due to the small number of ancestors [22] that comprise the base of this germplasm and to subsequent breeding and selection during cultivar development [23]. Wild species may have one or more positive alleles at major gene loci that influence agronomic traits [24]. Mining genes from wild relatives has proven successful in tomato [25], rice [26], and soybean [27–34].

Though exotic germplasm offers a vast genetic resource that can broaden soybean’s genetic base especially for disease and pest resistance [35], it has been difficult to select for yield improvement by targeting selection at the progeny derived from interspecific matings of elite cultivars with wild soybean accessions. A better approach is to reduce the genomic contribution of the wild soybean parent in any given progeny by utilizing one or more backcrosses, before selfing the resultant progeny lines to create backcross-derived inbred lines (BILs). This advanced backcross population approach has been proposed as a means to evaluate random chromosomal sections of the donor parent [such as wild soybean] in a genetic background that otherwise contains 75% (BC1) or even 87.5% (BC2) of the recurrent parent genome [36].

In soybean, molecular markers have been used extensively in recent decades to construct linkage and physical maps, and thereafter to identify and in some cases, confirm QTL for many agronomically important traits [37]. Soybean QTL studies that focused on root traits utilized crosses between *G*. *max* parental genotypes [5, 38, 39, 40]. But QTL alleles from exotic soybean germplasm have been reported by several researchers [34, 35, 41] to influence the seed yield in soybean. Informative markers flanking QTLs governing root-system architecture will facilitate marker-assisted selection of desirable root ideotypes. The objective of the present study was to identify QTLs for root architectural traits in an interspecific mapping population between *G*. *max* and its wild relative, *G*. *soja*.

## Materials and Methods

### Plant materials

**Fig 1:**
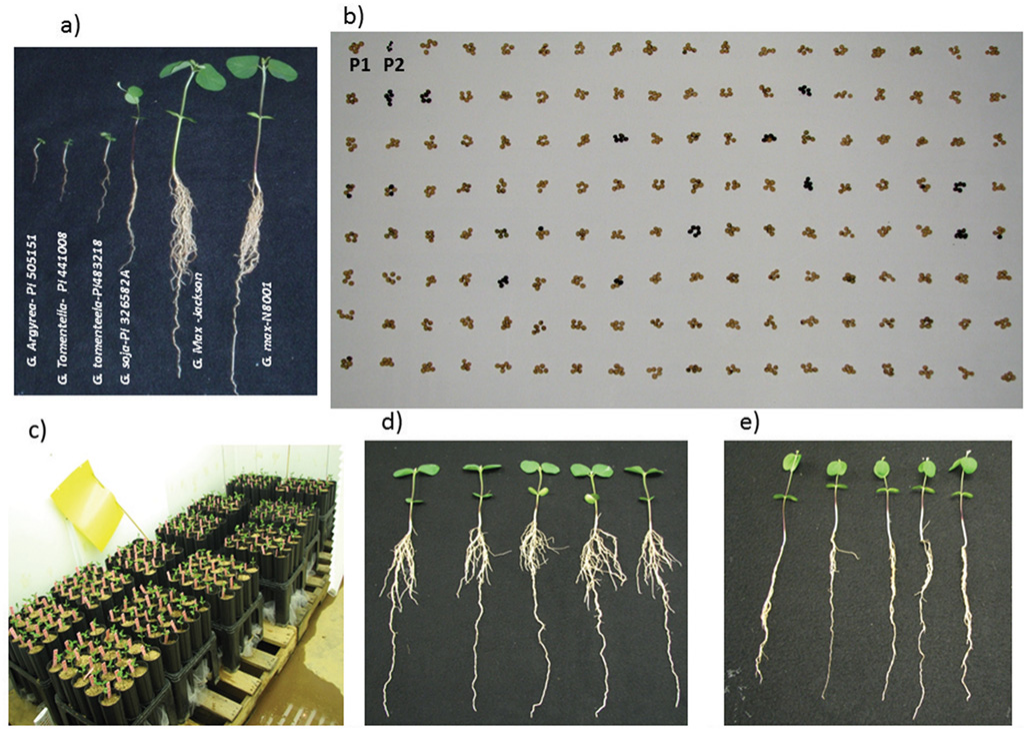
Variation for seed and plant architectural traits in soybean and the BIL population. a) Variation in root architecture in soybean accessions 12 days after sowing (das); b) Seed coat color of parents (Dunbar, P1 and PI 326582A, P2) and RILs c) Cone system for root screening d) Dunbar 12 das e) PI 326582A 12 das.

This study utilized a backcross-derived inbred line (BIL) mapping population, created by mating the *G*. *max* maturity group III soybean cv. ‘Dunbar’ (PI 552538) with a *G*. *soja* maturity group II so-called “wild” soybean accession (PI326582A). The phenotypic descriptors of the parental lines are presented in Table 1. The phenotypic variation of root traits in *G*. *soja*, PI326582A is shown in comparison to other soybean accessions in Fig. 1a. The segregation of seed size and color in Dunbar/PI326582A population is shown in Fig. 1b. The F_1_ plants were backcrossed to the Dunbar parent and the resulting 300+ BC_1_F_1_ plants were independently backcrossed again to the Dunbar parent to produce more than 300+ BC_2_F_1_ plants. Plant to progeny row (not single-seed descent) was used for generation advancement from the BC_2_F_1_ to the BC_2_F_4_ generation from which 296 BC_2_F_4.5_ progeny rows were separately harvested to produce F4-derived F5 inbred lines, henceforth referred to as BILs.

**Table 1:** Growth characteristics of the parental lines used in the study.

Trait	♀ parent Dunbar	♂ parent PI 326582A
Background	PI 552538 *Glycine max* (L.) Merr. FABACEAE (cultivated soybean) Platte x A3127	Plant introduction line *Glycine soja* Siebold & Zucc. FABACEAE (wild soybean)
Maturity group	MGIII	MGII
Stem term	indeterminate	indeterminate
Flower color	purple	purple
Hilum color	imperfect black	black
Pubescence color	gray	tawny
Seed coat color	yellow	black

### Phenotypic data collection

The genotypes were grown using the “cone" system [21] developed to study seedling root traits. The screening of the parental lines and 251 BILS were replicated six times in a randomized complete block design. The entire population was screened at the same time in a walk-in growth chamber that accommodated 76 racks (Stuewe and Sons, Oregon, USA) set on wooden pallets to facilitate support and water drainage (Fig. 1c). As *G*. *soja* exhibits considerable seed dormancy, the parental *G*. *soja* seed coat was scarified before planting by making a slanted cut in the seed coat at the opposite end of the embryo from the hilum to facilitate the germination process. A turface:sand (2:1 v/v) mixture (www.Hummert.com) was used as the growth medium [21]. The mixture is a growth medium similar to field soil and facilitates easy collection of the entire root system without damage [42]. Turface is calcined clay and has cation exchange capacity, so it is likely to have nutrients associated with its exchange sites. It adds water and air-holding pore space, prevents soil compaction, and allows better drainage. Sand facilitates the optimization of the air—water balance in the root zone. The media has no mechanical resistance to root penetration under well-watered conditions [21]. The seedlings were grown in cones to the V1 stage in a growth chamber with controlled conditions of 27/21°C day/night temperature, photoperiod of 16/8 day/night, 65% relative humidity, and an average light intensity of ~262 μmol m^-2^ s^-1^ measured at canopy level. At 12 days after sowing, intact seedlings were separated by cutting the cones longitudinally. The tap root length was measured with a ruler from the portion of the shoot that appeared white to the tip of the tap root. The length of the shoot was determined as that of the region above the white portion (either purple or green) up to the shoot apical meristem. The root system of each seedling was clipped and immediately washed in a tray of water, labeled, wrapped individually in wet paper towels and stored at 4°C for image analysis. Shoot dry weight was determined after shoot tissue (with cotyledons and leaves) had been dried in an oven at 65°C for 48 h.

### Root imaging and data analysis

The root systems of individual plants were scanned using a root scanner LA2400 coupled with WinRhizoPro software (Regent Instruments Inc., Canada). The images were analyzed using the WinRhizoPro software to count lateral roots. The phenotypic data was analyzed by the PROC MIXED procedure of SAS (version 8.2, SAS Institute, Inc., Cary, N.C, USA) with replicates and entries as random and fixed effects, respectively.

### QTL and candidate gene identification

An initial survey of 768 simple sequence repeats (SSR) and 243 single-nucleotide polymorphism (SNP) markers was performed with the Dunbar/PI 326582A parental lines. 312 polymorphic markers (103 SNPs, 205 SSR, and 4 classical loci {I/i, T/t, L1/l1, and L2/l2}) were identified. A genetic linkage map was constructed with 256 markers that align with the soybean consensus map 4.0 [43]. Some chromosomes have to be split owing to internal linkage gaps more than 50 Haldane cM (S1 Dataset). The information on molecular markers and their segregation pattern among 251 BILs are given in the S1 Dataset. These polymorphic markers spanned a genetic map distance of 2,118 cM. The mean chromosome length was 106 cM, with a mean genetic distance of 6.8 cM between any consecutive pair of mapped markers. Inclusive composite interval mapping was performed using ICI mapping software [44] to detect QTL and study the digenic epistatic QTL interaction. This software program uses an improved algorithm of composite interval mapping with increased power to detect QTLs, reduce false detection rates and have less biased QTL effect estimates. This is accomplished in two steps; stepwise regression followed by QTL scanning [44]. Based on the confidence interval spanning each major QTL identified in the study was compared to the QTLs in SoyBase [37] to determine whether any SoyBase-listed QTLs had positions within the same confidence intervals. In addition, candidate genes located within the confidence interval of the QTL peak were identified, extracted, and analyzed using the Soybean Knowledge Base (SoyKB) database [45]. DNA Sequence data of wild soybean accessions published [46, 47] were compared with Williams 82 reference sequence to identify single nucleotide variation for genes identified within the major QTL interval region. A meta-analysis of the candidate genes and their expression was performed using soybean Affymetrix expression data available in the Genevestigator database [48] and transcriptome dataset [49]. Based on these expression datasets, six genes were selected for quantitative real time PCR for studying the transcript abundance among parental lines and selected BILs (S1 Text) with different allele combination in major QTL region.

### RNA isolation and quantitative reverse- transcription (q-RT) polymerase chain reaction (PCR)

The selected BILs and parental lines were grown to the V1 stage and the root tissues were collected for RNA isolation. RNA was extracted from root tissues (100 mg tissues) using RNeasy Plant mini kit (Qiagen, CA, USA) according to manufacturer’s protocol. On-column DNA digestion was performed using RNase-Free DNase Set (Qiagen, CA, USA) according to the manufacturer’s protocol. Each sample (2μg of total RNA) was reverse-transcribed to cDNA in a 20μL reaction volume using RNA to cDNA EcopryTM Premix (Double primed) cDNA Synthesis Kit (Clontech, CA, USA). Quantitative RT-PCR (qRT-PCR) was performed using the cDNA product corresponding to 25 ng of total RNA in a 10μL reaction volume using Maxima SYBR Green/ROX qPCR Master Mix (2X) (Thermo, USA) on a ABI7900HT detection system (Applied BioSystems, Foster City, CA, USA). The expression data for each sample was generated from three biological and two technical replicates. The relative expression of the selected genes were expressed as mean standard deviation, in comparison to transcript abundance levels of ubiquitin, a housekeeping gene and analyzed using Delta Ct method [50]. The PCR conditions were as follows: 50°C for 2min, 95°C for 10 min, then 40 cycles of 95°C for 15 s, and 60°C for 1 min. To normalize the gene expression, ubiquitin (*Glyma20g27950*) was used as an internal control. All primers were designed using Primer3 web-interface (http://frodo.wi.mit.edu/primer3/ input.htm) [51] and the primer sequence information is given in S2 Text.

## Results

### Phenotypic variation among traits

**Fig 2:**
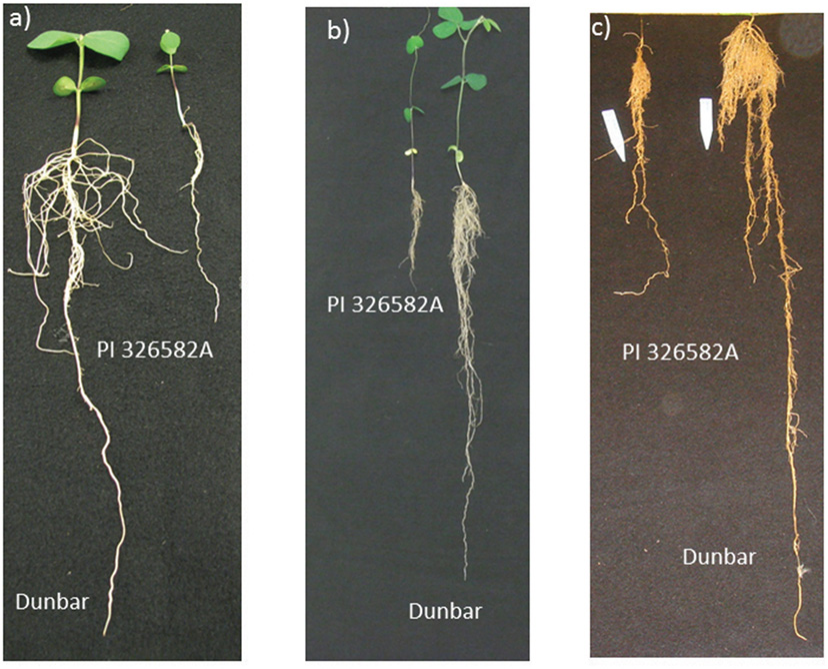
Root architecture contrasts between the two parents at: a) 12 days; b) 21 days; and c) 35 days after sowing.

**Fig 3:**
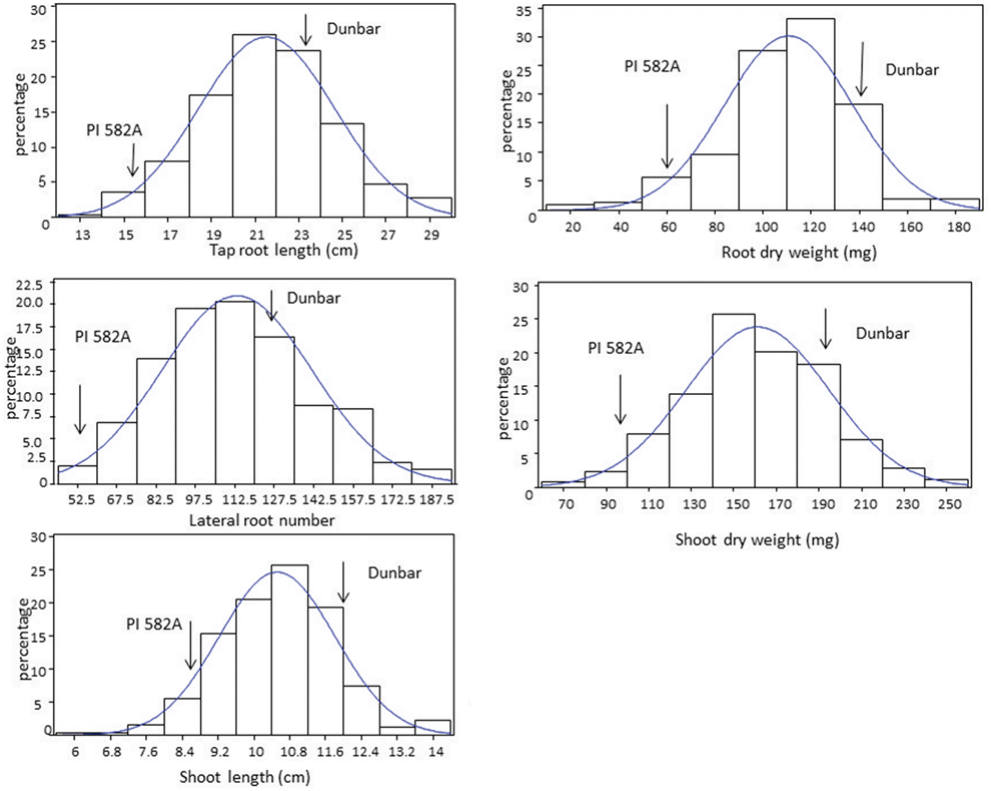
Distribution of means of 251 BILs for root and shoot traits. Parental values are indicated by arrows.

During the seedling stage (V1) and at 21 and 35 days after sowing (V3 and V4 stages, respectively) Dunbar showed a significantly longer tap root and higher lateral root number than PI 326582A (Fig. 1 d,e and Fig. 2; Table 2). The measured phenotypes of tap root length (TRL), lateral root number (LRN), shoot length (SL), shoot dry weight (SDW), and root dry weight (RDW) exhibited a normal distribution (Fig. 3), as confirmed by the application of the Shapiro—Wilk test. The respective coefficients of variation for the traits were TRL (17.0%), LRN (21.64%), SL (10.6%), SDW (5.04%), and RDW (8.85%). Dunbar, the cultivated soybean, showed the highest phenotypic values for shoot and root traits in comparison to the wild soybean, PI 326582A. However the backcross inbred lines (BILs) showed a transgressive segregation pattern for all traits reported with a higher range of phenotypic variation than either of the parental lines (Table 2).

**Table 2:** Statistical analyses of seedling root and shoot traits in the BIL population and the parents (n = 6) with a significant P value <.0001.

Traits	Parental lines Mean value			BIL (Number of lines: 251)	
	Dunbar	PI 326582A	Mid Parent	Mean± S.E	Range
TRL	23.8	16.8	20.3	21.5±1.3	12.25–29.22
LRN	126.00	50.67	88.3	113±10	49.33–192.83
SL	12.10	8.92	10.5	10.5±0.5	5.95–13.92
RDW	140.75	54.02	97.4	110.6±4.0	10.07–186.2
SDW	197.45	98.78	148.13	161.2±3.3	74.45–252.25

TRL: Tap root length (cm); LRN: Lateral root number; SL: Shoot length (cm); RDW: Root dry weight (mg); SDW: Shoot dry weight (mg).

### QTLs and their interaction

**Fig 4:**
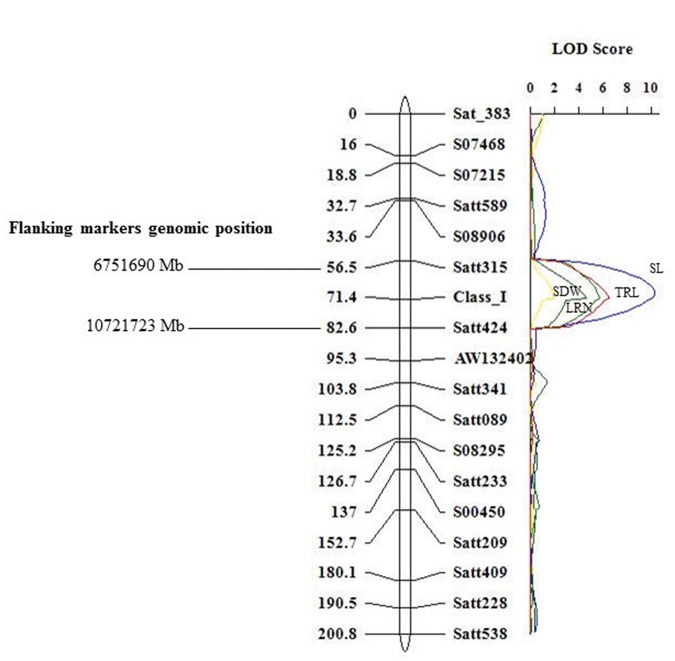
QTLs for root and shoot traits identified on chromosome 8 using inclusive composite interval mapping approach.

The use of interval mapping (IM) applied to the six measured shoot and root trait means led to the identification of five QTLs with their permutation-generated LOD score criteria for QTL significance declaration (Table 3). QTLs controlling TRL, LRN, SL and SDW were mapped on chromosome 8 within a confidence interval of 15 cM between Satt315 and the I locus (Table 3). The LOD score peak of each of these four QTLs exceeded 3.0 and accounted for from 4.6 to 10.3 percent of phenotypic variation (Table 3, Fig. 4). With respect to the significant QTLs identified on chromosome 8 for TRL, LRN, SL and SDW, the allele conferring greater length and number was contributed by the cultivated soybean parent, Dunbar (Table 3). The QTL analysis also identified a SDW QTL on chromosome 12 (Table 3) and no significant QTL with LOD score >3.0 was detected for RDW. However, the interaction analysis identified QTLs for RDW involving five chromosomal regions with (Chromosomes 8 and 12) without (Chromosomes 1, 6, and 10) main effects, with positive alleles contributed by Dunbar. Both these chromosomal regions on chromosomes 8 and 12 have additive effects on RDW individually and have negative effect on interaction (Table 4). Thus the RDW QTL in this mapping population might be controlled by polygenes or QTLs with minor effects. However, the similar chromosomal regions of chromosomes 8 and 12 showed higher additive effects for the shoot length trait.

**Table 3:** Quantitative trait loci (QTL) for root and shoot architecture traits and their additive effects identified by inclusive composite interval mapping approach using ICI Mapping software.

Trait	Chromosome	QTL peak Position (cM)	Left Marker	Right Marker	ThresholdLOD	LOD	PVE (%)	Additive effect
TRL	8	71	Satt315	class I	3.09	6.52	12.28	1.87
LRN	8	71	Satt315	class I	3.22	5.72	11.04	16.13
SL	8	69	Satt315	class I	3.21	10.27	20.77	1.01
SDW	8	71	Satt315	class I	3.04	4.61	8.49	16.74
	12	16	Satt253	Satt142		3.54	7.77	23.34

TRL: Tap root length (cm); LRN: Lateral root number; SL: Shoot length (cm); SDW: Shoot dry weight (mg); cM: Centi Morgan

**Table 4:** Epistatic QTLs identified for root dry weight with LOD value < 5.0 in Dunbar/PI 326582A population.

Chromosome	Marker interval	AE^+^	Chromosome	Marker interval	AE^+^	PVE (%)	AA interaction
1c	Satt408-Satt129	22.18	6b	Sat_062-Satt281	23.44	10.8	-25.82
6b	Satt281-Satt457	21.27	8	S08906-Satt315	22.36	12.6	-26.68
8	S08906-Satt315	15.71	10b	Satt592-Satt331	20.01	9.8	-19.76
6b	Satt281-Satt457	22.15	12b	Satt142-Satt434	23.32	11.5	-27.25
8	S08906-Satt315	21.77	12b	Satt142-Satt434	23.53	10.7	-25.23

AE: Additive effect; PVE: Phenotypic variation explained in per cent; AA: Additive x Additive interaction

^+^Positive value indicate that the Dunbar allele increase the phenotypic value.

**Fig 5:**
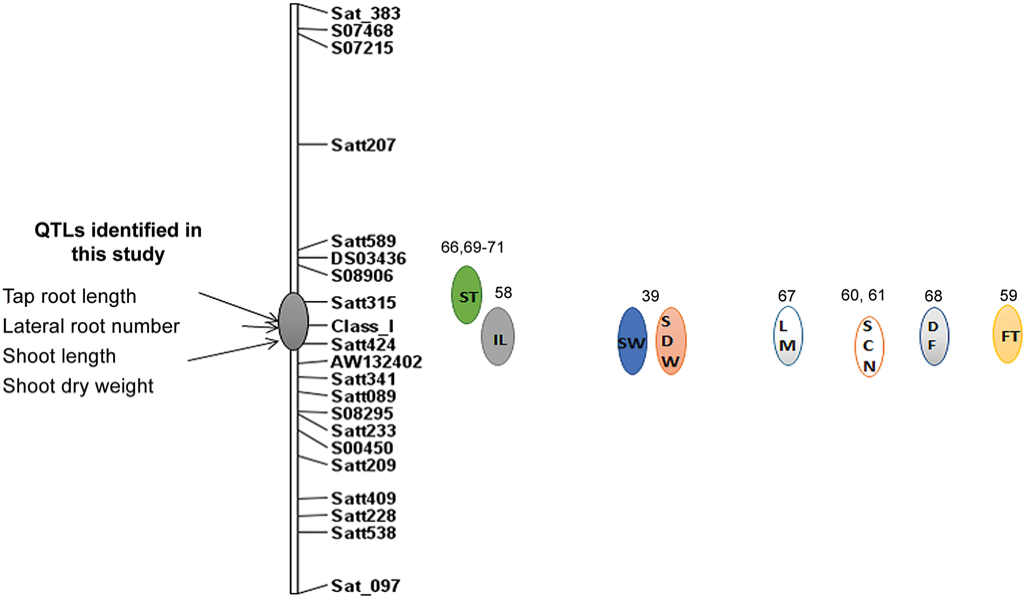
Co-location of other plant and seed morphology traits in the identified candidate QTL region on chromosome 8.

In Soybase, the markers flanking the root QTL regions identified on chromosome 8 were also associated with several other agronomic traits in soybean. The flanking marker Satt315 of this region was also associated with QTL for seed length, row spacing response, seed isoflavone components, and several other agronomic traits (Fig. 5). The Satt424 locus near the locus I was positively associated with map positions for internode length, hypocotyl length, and shoot dry weight. The QTL map location for root and shoot traits (Table 3) near the I locus on chromosome. 8 is also closely linked to a gene for soybean cyst nematode (SCN) resistance/susceptibility.

### Candidate genes located within QTL interval on chromosome 8

**Fig 6:**
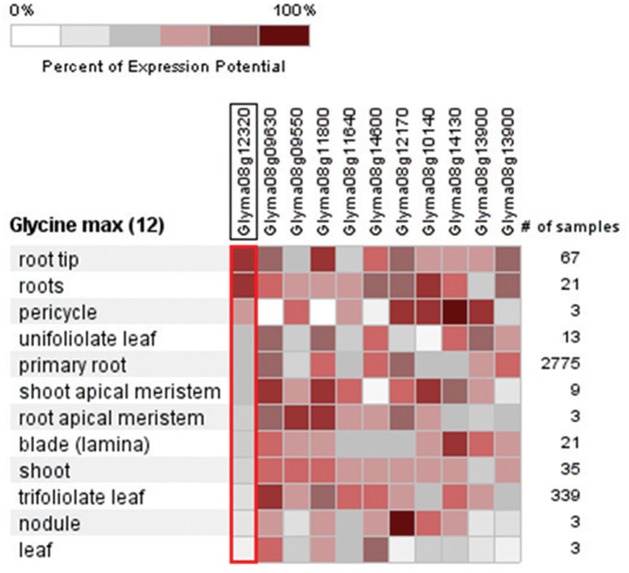
Candidate genes identified on QTL region on chromosome 8 and their expression pattern in 12 soybean tissues derived from Affymetrix gene chip data in Genevestigator software.

**Fig 7:**
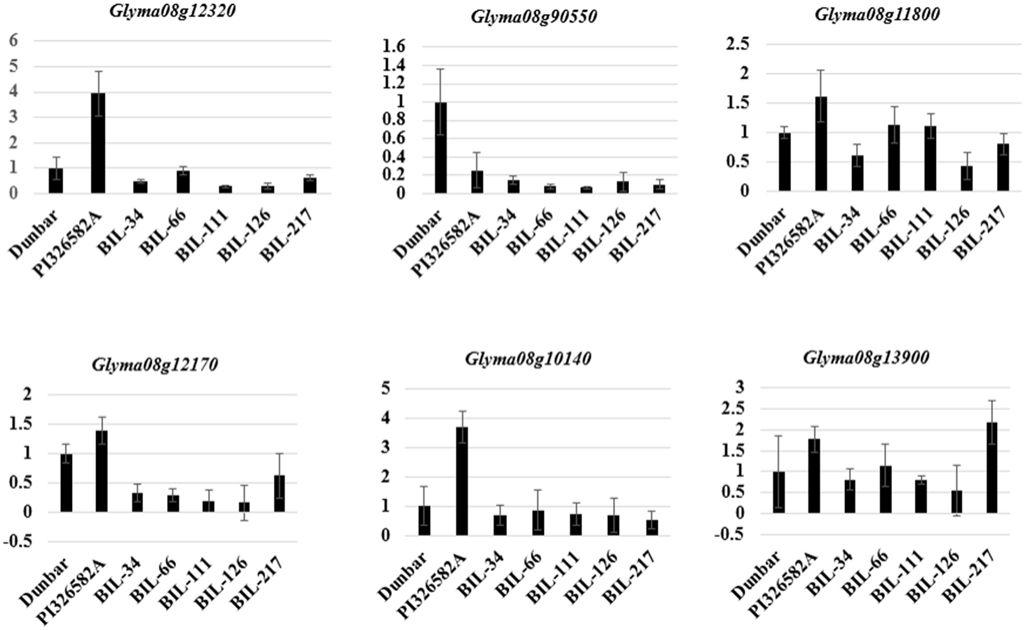
Differential expression of key transcription factors identified in the QTL interval on chromosome 8.

Based on comparison of the genetic map with the sequence map of Williams82 (*Glyma* 1.0 version) in SoyBase the marker flanking the QTL on chromosome 8, Satt315 and Satt424 were located between 6751690 and 6751725 and between 10721723 to 10721881 Mb, respectively. Within this genomic interval region, 504 genes were identified from Williams82 and sorted based on the expression pattern levels in soybean root and nodule tissues (S2 Dataset). Based on Soybase transcriptome data only 75 out of 504 genes showed higher and tissue specific expressions in soybean root and nodules (S3 Text). The nuclear transport factor 2 (*Glyma08g11070*) showed more than a 300-fold increased expression in root tissues. Although 12 transcription factor (TF) families were identified in this QTL interval, only a few TFs including Homeobox domain (*Glyma08g14130*), GRAS (*Glyma08g10140*), WRKY (*Glyma08g12460*) and bZIP (*Glyma08g12170*) showed more than a 50- fold expression in soybean root tissues (S3 Text). Based on Affymetrix gene chip data, six Transcription factors (MYB HD; *Glyma08g12320*, TPR: *Glyma08g09550*, C2H2 Zn: *Glyma08g11800*, BZIP: *Glyma08g12170*, GRAS: *Glyma08g10140* and Ring finger: *Glyma08g13900*) only showed higher expression on soybean root tissues (Fig. 6). All six TFs showed a higher expression in the wild soybean parental line, PI326582A than the Dunbar (Fig. 7), except for TPR (*Glyma08g09550*) transcription factor. Even the BIL126 with wild soybean parental allele in the QTL region did not showed similar expression as wild soybean parent. All the selected BILs showed expression level of TFs within the parental values except in BIL 217 for Ring finger TF (Fig. 7).

Based on public soybean Affymetrix expression data, two TFs, AP2-EREBP (*Glyma08g14600*) and bZIP (*Glyma08g12170*) showed higher expression in root tissues (Fig. 6). This AP2 TF also showed higher expression in root tissues (37- fold) and in nodules (13- fold) within the transcriptome data (S3 Text). Key cell wall expansion-related genes (*Glyma08g11300* and *Glyma08g09940*) encoding xyloglucan endo-transglycosylases (XET) with high expression in both root and shoot tissues were identified. However, the gene *Glyma08g11300* showed a higher expression in root tissues than *Glyma08g09940*.

### Genomic variation between cultivated and wild soybeans in the QTL interval

For the genomic comparison of the QTL region of chromosome 8, the DNA sequence data of Williams 82 (cultivated soybean) was compared with 17 and 2 diverse wild soybean accessions from China and Korea, respectively. These accessions showed conserved single nucleotide polymorphic (SNP) variation within them (S3 Dataset) in comparison to the cultivated soybean reference genome, Williams 82. Among the two Korea wild soybean accessions, PI 407162 sequenced at 15X depth by our lab also enabled us to identify non-synonymous SNPs in genes located within the QTL interval. Among the 504 genes identified within this QTL interval, 43 genes had non-synonymous SNPs that altered the amino acid (S4 Dataset), resulting in changing the translated protein. Most of these genes showed conserved SNP variation among diverse wild soybeans accessions from China and Korea. 12 genes identified with a non-synonymous SNP also showed higher expression in soybean root and nodule tissues (S3 Text). In particular, two transcription factors like Homeobox domain (*Glyma08g14130*) and C3H type 1 (*Glyma08g09630*) showed high expression in root tissues in both Affymetrix (Fig. 6) and transcriptome (S3 Text) public datasets.

## Discussion

### Root QTL mapping in soybean

Our study is the first to utilize an interspecific mating (*G*. *max* × *G*. *soja*) to create a BC2F4-derived population of BILs to map QTLs for root traits. Other studies [5, 39–40] used only intra-species (*G*. *max* × *G*. *max*) matings. In our mapping population, the interpretation of the range in the BIL phenotypes was likely confounded by the BC2-mediated skewing of the Dunbar AA genotypes vs. PI aa genotypes to a 7:1 ratio at one locus, but with two loci, the expected ratio of the genotypes is 49AABB:7AAbb:7aaBB:1aabb. As Dunbar is the “high” parent for all traits studied, the BILs with Dunbar AABB BIL homozygotes are more frequent (S1 Dataset). The root and shoot QTLs identified in the present study were identified using a well-watered cone system in a growth chamber. We do not know whether the measured additive effect in the BILs with respect to the contrasting parental alleles at each QTL would be the same or different in a less optimal water scenario.

However, for several root traits detected between Williams 82 and a soybean breeding line, genetic variation was reported to enable drought avoidance and yield advantage [14]. Soybean plants with deep rooting ability [52, 53] and more fibrous roots [18] are supposed to offer the inherent advantage of acquiring water more efficiently than shallow-rooted genotypes: a test of that supposition has yet to be realized by the release of an elite cultivar with these traits. In the present study, we discovered that soybean chromosome 8 (previously linkage group A2) harbored several QTLs including tap root length, shoot length, and lateral root number. Epistatic QTLs were detected for root dry weight involving five different chromosomal regions, denoting that this trait might be controlled by polygenes. These findings are corroborated by recent studies in soybean root studies at the seedling stage [54–56] and in matured plants under field conditions [57]. This region on chromosome 8 interacts with chromosome 12 region and negatively affects the root dry weight. Similar negative interactions of additive QTLs have been reported for root weight in seedling-stage root-mapping studies in soybean [56]. The QTL for tap root length (Satt315-Satt424) was located in confidence intervals that were close to peak LOD scores exhibited by QTLs for maximum root length, root weight, and tap root length in another study [40]. The aggregation of QTLs around markers Satt315—I locus—Satt424 on chromosome 8 for root and shoot traits indicates a positive relationship exists between them, and also points to a candidate region governing early seedling vigor in soybean.

### Genomic regions for improving abiotic and biotic stress tolerance

The QTLs identified in this study on chromosome 8 influenced both shoot and root growth. It is of interest to compare the markers associated with our QTL with QTLs that have been reported earlier. For example, SSR marker Satt424 associated with TRL and SL in this study was, in a prior study, associated with a QTL that explained 46% of variation for internode length [58], and in another study was associated with a large-effect QTL conferring flooding tolerance [59]. In addition, this region confers resistance to biotic stresses such as soybean cyst nematode (QTL SCN30–3 [60]; SCN29–5 [61], and *Sclerotinia* root rot (QTL Sclero 2–2, 3–2, 5–1, 6–2) [62]. The QTL map location for root and shoot traits (Table 4) near the class I locus, which controls seed coat pigmentation [63] on chromosome 8, is closely linked to Rhg4 gene [64] that encodes serine hydroxylmethyltransferase and confers resistance to soybean cyst nematode [65]. A QTL for soybean seed length was also flanked by this marker [66]. Collectively, the marker interval Satt315–Satt424 contains QTLs for growth and yield components including leaf width, leaf shape [67]; days to flowering [68]; seed weight [69,70]; pod maturity and seed protein content [71] in Soybase [37]. These loci may be investigated as candidates for utilization in marker assisted breeding (MAB) programs to improve root architecture and other traits.

Based on this study, using an inter-specific backcross population, the observed phenotypes and the QTL analyses indicate that the cultivated allele was superior to the wild allele for all root architectural traits. A similar difference in allelic effects between cultivated and wild accession has been reported for domestication-related traits in soybean [72]. However another recent study in an inter-specific mapping population [73] identified QTLs for root traits, total root length, and surface area with positive alleles contributed by a wild soybean accession, PI 407162.

### Key candidate gene underlying QTL on chromosome 8

Among the eleven TFs identified within the QTL interval, the MYB TF (*Glyma08g11640*) showed higher expression in shoot tissues (shoot apical meristem and trifoliate leaves) (Fig. 6). But the MYB-HD TF showed a higher expression in root tissues (root, root tip, and pericycle) in both the Affymetrix gene chip expression data (Fig. 6) and the Soybase RNA sequencing data (S3 Text). This TF (*Glyma08g12320*) also showed a higher expression in wild soybean accession PI407162 and none of the interspecific BILs showed an expression pattern as high as that of the parental lines (Fig. 7). The allelic interaction between cultivated and wild soybean may have affected the gene expression in BILs. Similar distinct expression patterns of root related genes were found in a soybean inter-specific mapping population [73]. Role of MYB TFs in root formation were reported earlier in soybean and *Arabidopsis* [74]. In these datasets, (S3 Text; Fig. 6) the AP2 TF as being highly expressed in shoot, root, and nodule tissues. The role of this TF in nodulation and association with the Nod factor signaling pathway was shown earlier in *Medicago truncatula* [75] and its over-expression also confers drought tolerance in *Arabidopsis* [76]. Interestingly, the bZIP TF showed a higher expression in root pericycle and nodules in both datasets. The pericycle cells are key cell types that form lateral root primordium, which decides the lateral root number. A similar root-specific bZIP TF has been reported to be responsive during water stress and involved in intracellular signaling in both tepary and common beans [77]. This TF was also reported to be involved in controlling nodule number by early initiation of nodulation in *Lotus japonicus* [78]. Drought-tolerant soybean also showed high nodule number and size under water-deficit conditions [79,80], resulting in better nitrogen fixation ability that translated into higher yield in drought stress conditions [81]. However, more research is needed to illuminate the role of this TF in increasing nodule number and sustaining nitrogen fixation capacity under drought conditions. Among the six TFs selected based on their Affymetrix gene chip expression pattern, TPR transcription factor showed a higher expression in cultivated soybean, Dunbar. Similar expression pattern for a TPR TF in the same gene family was reported in another cultivated soybean, V71–370 with non-synonymous SNPs in comparison to a wild soybean, PI407162 [73]. A similar class of transcription factor was expressed in soybean roots as an early response to iron availability [82]. Among the 42 genes identified with non-synonymous SNPs (S4 Dataset) in wild soybean accessions, only two TFs (C3H type 1 and Homeobox Domain) that showed higher expression in both Affymetrix and trancriptome datasets. These TFs might be the possible candidates for downstream genes associated with root system architecture in soybeans. An association of SNP variants with the root phenotype must be established in the future to identify genes that regulate root development in soybean. The conserved SNPs among diverse wild soybean accessions (S3 Dataset and S4 Dataset) might be best choices for studying the evolution of root traits in cultivated soybean.

## Conclusion

This study aimed to identify quantitative trait loci associated with root and shoot growth at the seedling stage in soybean. A major locus was identified on chromosome 8 flanked by markers Satt315 and Class I spanning a 15 cM region. The beneficial alleles for all the studied traits were contributed by the Dunbar parent. The BILs with deeper root system than the Dunbar recurrent parent will be tested for root traits and their contribution to productivity in water-limited/rainfed environments. The development of near-isogenic lines containing these candidate regions is also in progress, with the goal of elucidating the biological value of these alleles under field conditions.

## Supporting Information

S1 Dataset Chromosome-wise marker segregation pattern among 251 BILs of Dunbar x PI326582A population (XLSX)

S2 Dataset List of 504 genes located in the QTL confidence interval on chromosome 8 (XLSX)

S3 Dataset Conserved SNP variation among diverse wild soybean accessions within genomic QTL interval on chromosome 8 (XLSX)

S4 Dataset List of genes identified within the QTL region on chromosome 8 with non-synonymous SNPs in wild soybean accessions. (XLSX)

S1 Text BILs selected for qRT-PCR gene expression analysis based on chromosome 8 QTL region (DOCX)

S2 Text Primer sequence of genes selected for qRT-PCR analysis (DOCX)

S3 Text List of genes identified in QTL confidence interval and showing high and tissue specific expression in root tissues in SoyBase database (DOCX)
