# Supplementary material for: Identification of Novel QTL Governing Root Architectural Traits in an Interspecific Soybean Population
Source: PLoS One. 2015 Mar 10;10(3):e0120490. doi: 10.1371/journal.pone.0120490 (PMC4355624; doi:10.1371/journal.pone.0120490)
Supplement: S1 Text — (DOCX) [file pone.0120490.s005.docx]

S1 Text. BILs selected for qRT-PCR gene expression analysis based on chromosome 8 QTL region

| Flanking markers | BIL#34 | BIL#66 | BIL#111 | BIL#126 | BIL#217 |
| --- | --- | --- | --- | --- | --- |
| Satt315 | A | A | A | B | A |
| Class I | A | A | A | B | A |
| Satt424 | A | H | A | B | A |

A: Dunbar allele; B: PI326582A allele
