# Supplementary material for: Identification of Novel QTL Governing Root Architectural Traits in an Interspecific Soybean Population
Source: PLoS One. 2015 Mar 10;10(3):e0120490. doi: 10.1371/journal.pone.0120490 (PMC4355624; doi:10.1371/journal.pone.0120490)
Supplement: S2 Text — (DOCX) [file pone.0120490.s006.docx]

S2 Text. Primer sequence of genes selected for qRT-PCR analysis

| **Gene ID** | **Sequence information** |
| --- | --- |
| Glyma08g12320-F | GTTCAAATAGTGGGTCGCAAC |
| Glyma08g12320-R | TTCCGAAGTTCAACTCGCTAG |
| Glyma08g09550-F | CTCTTTCGAGGCGCTCTTC |
| Glyma08g09550-R | ATGAGCTTTACGTCTCTGGC |
| Glyma08g11800-F | GACAGAGTTGGTTCCTTGAGAG |
| Glyma08g11800-R | GCAGAGTCTAGTTCAGATCCAG |
| Glyma08g12170-F | GGAACAGAGGAAGAGGAAGAG |
| Glyma08g12170-R | GTGTTCATTTGGGTGTTCTCC |
| Glyma08g10140-F | GTTCGTTTCGACCGGATTTTC |
| Glyma08g10140-R | CCCAACATGAGACAACCATTG |
| Glyma08g14130-F | CGCAGTACGTAAAGATGGAGG |
| Glyma08g14130-R | CTTCCTCTTAACTCCATTCCTCTG |
| Glyma08g13900-F | GGTTTGCCCGATTTGTCTTAC |
| Glyma08g13900-R | TCTGGTAGTGATTGTGCTTCG |
| Glyma20g27950-F | AATCAACCCTTCACCTCGTC |
| Glyma20g27950-R | CTCCTTGTCCTGAATCTTAGCC |
