# Supplementary material for: Identification of Novel QTL Governing Root Architectural Traits in an Interspecific Soybean Population
Source: PLoS One. 2015 Mar 10;10(3):e0120490. doi: 10.1371/journal.pone.0120490 (PMC4355624; doi:10.1371/journal.pone.0120490)
Supplement: S3 Text — (DOCX) [file pone.0120490.s007.docx]

S3 Text. List of genes identified in QTL confidence interval and showing high and tissue specific expression in root tissues in SoyBase database

| Gene ID  (Annotation) | Young  leaf | flower | one cm pod | pod shell 10DAF | pod shell 14DAF | seed 10DAF | seed 14DAF | seed 21DAF | seed 25DAF | seed 28DAF | seed 35DAF | seed 42DAF | root | nodule |
| --- | --- | --- | --- | --- | --- | --- | --- | --- | --- | --- | --- | --- | --- | --- |
| *Glyma08g11070*  (Nuclear transport factor 2) | 128 | 149 | 100 | 95 | 106 | 42 | 108 | 88 | 104 | 69 | 99 | 58 | 386 | 114 |
| *Glyma08g12140*  (Tubulin family) | 202 | 99 | 226 | 178 | 177 | 45 | 116 | 175 | 127 | 80 | 103 | 75 | 247 | 60 |
| *Glyma08g11490*  (Serine hydroxymethyl transferase) | 107 | 51 | 132 | 158 | 201 | 12 | 53 | 48 | 37 | 18 | 22 | 18 | 226 | 67 |
| *Glyma08g14130*  (Homeobox domain TF) | 105 | 121 | 123 | 127 | 58 | 39 | 36 | 34 | 40 | 17 | 50 | 35 | 173 | 59 |
| *Glyma08g10970*  (unknown function) | 97 | 56 | 59 | 52 | 56 | 17 | 40 | 32 | 29 | 13 | 20 | 9 | 105 | 59 |
| *Glyma08g10140*  (GRAS TF) | 32 | 48 | 20 | 20 | 27 | 7 | 19 | 19 | 21 | 13 | 23 | 17 | 76 | 40 |
| *Glyma08g12460*  (WRKY TF) | 1 | 1 | 1 | 1 | 0 | 0 | 0 | 0 | 0 | 0 | 1 | 0 | 76 | 29 |
| *Glyma08g11000*  (unknown function) | 52 | 36 | 42 | 36 | 28 | 1 | 2 | 7 | 4 | 1 | 1 | 1 | 74 | 29 |
| *Glyma08g10010*  (Mandelate racemase) | 2 | 13 | 34 | 29 | 64 | 18 | 21 | 10 | 3 | 1 | 1 | 0 | 69 | 29 |
| *Glyma08g13300*  (GDP dissociation inhibitor) | 27 | 33 | 52 | 53 | 47 | 16 | 29 | 24 | 26 | 17 | 30 | 17 | 67 | 26 |
| *Glyma08g12880*  (Sulfite transporter) | 0 | 0 | 0 | 0 | 0 | 0 | 0 | 0 | 0 | 0 | 0 | 0 | 55 | 2 |
| *Glyma08g12170*  (BZip TF) | 14 | 34 | 15 | 22 | 15 | 1 | 4 | 2 | 3 | 1 | 6 | 2 | 54 | 268 |
| *Glyma08g12560*  (NB-ARC domain) | 0 | 1 | 0 | 0 | 0 | 0 | 0 | 0 | 0 | 0 | 0 | 0 | 51 | 1 |
| *Glyma08g09520*  (unknown function) | 30 | 22 | 29 | 32 | 37 | 23 | 24 | 19 | 31 | 21 | 22 | 16 | 50 | 48 |
| *Glyma08g09740*  (Endomembrane protein) | 30 | 39 | 39 | 36 | 35 | 19 | 29 | 33 | 17 | 13 | 24 | 16 | 48 | 32 |
| *Glyma08g14230*  (Ras related GTPASE) | 7 | 25 | 6 | 8 | 11 | 3 | 4 | 8 | 8 | 7 | 8 | 5 | 44 | 17 |
| *Glyma08g14600*  (AP2-EREBP TF) | 1 | 3 | 0 | 1 | 2 | 1 | 3 | 2 | 1 | 1 | 0 | 1 | 37 | 13 |
| *Glyma08g11980*  (AMP binding protein) | 3 | 11 | 3 | 2 | 1 | 5 | 8 | 12 | 8 | 3 | 3 | 3 | 33 | 5 |
| *Glyma08g12220*  (14-3-3 protein) | 32 | 23 | 30 | 24 | 25 | 13 | 26 | 23 | 22 | 12 | 16 | 12 | 32 | 33 |
| *Glyma08g11640*  (MYB TF) | 15 | 16 | 15 | 13 | 9 | 4 | 8 | 8 | 10 | 5 | 11 | 10 | 30 | 42 |
| *Glyma08g11800*  (C2H2 ZN TF) | 17 | 21 | 18 | 16 | 8 | 8 | 10 | 14 | 8 | 5 | 12 | 7 | 28 | 16 |
| *Glyma08g10950*  (Cytochrome p450) | 0 | 8 | 4 | 1 | 1 | 0 | 0 | 0 | 0 | 0 | 2 | 5 | 28 | 1 |
| *Glyma08g12550*  (potassium channel tetramerisation domain) | 14 | 16 | 14 | 10 | 10 | 2 | 7 | 11 | 15 | 8 | 12 | 10 | 27 | 15 |
| *Glyma08g11410*  (Rapid alkanization factor) | 2 | 3 | 2 | 3 | 3 | 0 | 1 | 0 | 0 | 0 | 0 | 0 | 26 | 3 |
| *Glyma08g13470*  (transmembrane emp24 domain) | 15 | 21 | 20 | 19 | 14 | 19 | 28 | 24 | 15 | 10 | 14 | 8 | 25 | 16 |
| *Glyma08g13980*  (Eukaryotic initiation factor 4B) | 18 | 17 | 18 | 12 | 8 | 4 | 9 | 12 | 11 | 4 | 9 | 5 | 24 | 15 |
| *Glyma08g14080*  (Clathrin assembly protein) | 8 | 7 | 4 | 3 | 10 | 1 | 2 | 2 | 1 | 0 | 2 | 3 | 24 | 11 |
| *Glyma08g11300*  Xyloglucan endo-transglycolase (XET) | 3 | 21 | 9 | 10 | 9 | 4 | 7 | 7 | 11 | 5 | 4 | 1 | 23 | 10 |
| *Glyma08g13990*  (GDSL like lipase) | 15 | 9 | 12 | 9 | 6 | 3 | 5 | 11 | 4 | 2 | 3 | 2 | 22 | 23 |
| *Glyma08g09730*  (sodium/hydrogen exchanger) | 2 | 5 | 4 | 3 | 2 | 1 | 1 | 1 | 2 | 2 | 4 | 4 | 21 | 1 |
| *Glyma08g09940*  (XET) | 7 | 5 | 6 | 8 | 6 | 6 | 9 | 9 | 11 | 5 | 10 | 6 | 20 | 33 |
| *Glyma08g10680*  (F box/Leucine rice repeat protein) | 9 | 11 | 9 | 10 | 7 | 2 | 7 | 6 | 9 | 4 | 12 | 9 | 20 | 14 |
| *Glyma08g14720*  (Aminotransferases) | 9 | 7 | 11 | 8 | 6 | 3 | 5 | 5 | 3 | 3 | 2 | 2 | 20 | 4 |
| *Glyma08g13910*  (unknown function) | 4 | 10 | 5 | 5 | 3 | 2 | 3 | 5 | 3 | 2 | 6 | 4 | 19 | 12 |
| *Glyma08g13620*  (Domain of unknown function) | 0 | 0 | 0 | 0 | 0 | 0 | 0 | 0 | 0 | 0 | 0 | 0 | 19 | 2 |
| *Glyma08g09870*  (Phosphatase family) | 0 | 0 | 0 | 0 | 0 | 0 | 0 | 0 | 0 | 0 | 0 | 0 | 19 | 0 |
| *Glyma08g11100*  (Domain of unknown function) | 8 | 8 | 7 | 5 | 4 | 1 | 2 | 1 | 1 | 0 | 0 | 0 | 18 | 0 |
| *Glyma08g09550*  (TPR TF) | 15 | 8 | 10 | 13 | 12 | 3 | 7 | 8 | 7 | 3 | 4 | 3 | 17 | 33 |
| *Glyma08g12520*  (Glutathione S transferase) | 2 | 2 | 2 | 4 | 6 | 1 | 1 | 1 | 1 | 1 | 2 | 2 | 17 | 1 |
| *Glyma08g09880*  (Calcineurin like phosphoesterase) | 0 | 1 | 0 | 0 | 0 | 0 | 0 | 0 | 0 | 0 | 0 | 0 | 16 | 1 |
| *Glyma08g13260*  (D-mannose binding lectin) | 0 | 0 | 0 | 0 | 0 | 0 | 0 | 0 | 0 | 0 | 0 | 0 | 16 | 0 |
| *Glyma08g12290*  (Protein kinase domain) | 5 | 2 | 1 | 2 | 1 | 0 | 1 | 1 | 3 | 1 | 2 | 2 | 15 | 2 |
| *Glyma08g12120*  (Karyopherin beta 3) | 5 | 11 | 6 | 7 | 5 | 2 | 3 | 4 | 5 | 3 | 6 | 4 | 14 | 9 |
| *Glyma08g09680*  (Major facilitator family) | 7 | 10 | 7 | 7 | 7 | 3 | 8 | 6 | 3 | 2 | 3 | 2 | 14 | 8 |
| *Glyma08g14310*  (Leucine rice repeat protein) | 4 | 5 | 4 | 5 | 4 | 1 | 3 | 1 | 3 | 1 | 4 | 3 | 14 | 4 |
| *Glyma08g10130*  (unknown function) | 8 | 9 | 8 | 6 | 6 | 11 | 9 | 6 | 6 | 3 | 4 | 3 | 13 | 9 |
| *Glyma08g09630*  (C3H type 1 TF) | 9 | 11 | 8 | 10 | 5 | 3 | 5 | 9 | 7 | 3 | 8 | 5 | 13 | 8 |
| *Glyma08g13810*  (Actin) | 7 | 5 | 7 | 8 | 8 | 2 | 3 | 2 | 3 | 1 | 3 | 3 | 13 | 6 |
| *Glyma08g09960*  (unknown) | 3 | 10 | 6 | 5 | 4 | 3 | 3 | 4 | 3 | 2 | 5 | 3 | 13 | 4 |
| *Glyma08g12490*  (unknown) | 0 | 1 | 1 | 2 | 1 | 0 | 0 | 0 | 1 | 0 | 1 | 0 | 13 | 1 |
| *Glyma08g14500*  (TPR TF) | 6 | 5 | 7 | 8 | 7 | 2 | 5 | 6 | 5 | 2 | 4 | 2 | 12 | 1 |
| *Glyma08g09640*  (unknown function) | 7 | 14 | 10 | 9 | 7 | 6 | 7 | 10 | 9 | 3 | 6 | 5 | 11 | 23 |
| *Glyma08g09930*  (unknown) | 7 | 8 | 8 | 9 | 8 | 2 | 4 | 3 | 7 | 3 | 7 | 6 | 11 | 8 |
| *Glyma08g12050*  (unknown) | 8 | 6 | 6 | 7 | 7 | 3 | 7 | 7 | 7 | 4 | 9 | 3 | 11 | 4 |
| *Glyma08g13240*  (LETM1 like protein) | 6 | 6 | 7 | 9 | 5 | 2 | 5 | 5 | 5 | 3 | 6 | 3 | 10 | 7 |
| *Glyma08g10040*  (unknown function) | 2 | 7 | 9 | 9 | 5 | 8 | 7 | 7 | 5 | 3 | 7 | 4 | 10 | 1 |
| *Glyma08g13640*  (Amino acid permease) | 1 | 4 | 5 | 4 | 6 | 2 | 1 | 1 | 0 | 0 | 0 | 0 | 10 | 0 |
| *Glyma08g13900*  (Ring finger TF) | 0 | 2 | 0 | 0 | 1 | 0 | 0 | 0 | 0 | 0 | 0 | 0 | 10 | 0 |
| *Glyma08g10750*  (Cation efflux family) | 4 | 5 | 5 | 3 | 3 | 1 | 4 | 6 | 4 | 3 | 8 | 6 | 9 | 7 |
| *Glyma08g11970*  (Bacterial transferase hexapeptide) | 6 | 5 | 6 | 6 | 5 | 3 | 5 | 4 | 4 | 2 | 4 | 4 | 8 | 10 |
| *Glyma08g14170*  (integral membrane protein) | 5 | 5 | 4 | 4 | 3 | 1 | 3 | 2 | 3 | 1 | 3 | 2 | 8 | 4 |
| *Glyma08g12320*  (MYB-HD TF) | 0 | 2 | 1 | 1 | 1 | 0 | 0 | 0 | 0 | 0 | 0 | 0 | 8 | 1 |
| *Glyma08g12910*  (unknown) | 1 | 1 | 1 | 1 | 1 | 0 | 0 | 0 | 0 | 0 | 0 | 0 | 8 | 0 |
| *Glyma08g12090*  (unknown function) | 4 | 4 | 4 | 4 | 2 | 2 | 3 | 3 | 3 | 2 | 4 | 2 | 7 | 5 |
| *Glyma08g13340*  (Magnesium transporter) | 2 | 3 | 3 | 2 | 1 | 1 | 2 | 3 | 1 | 0 | 1 | 1 | 7 | 3 |
| *Glyma08g14520*  (sugar transporter) | 0 | 1 | 0 | 0 | 0 | 0 | 0 | 0 | 0 | 0 | 0 | 0 | 7 | 2 |
| *Glyma08g14700*  (Sulfate transporter) | 0 | 4 | 2 | 1 | 2 | 0 | 0 | 0 | 0 | 0 | 0 | 1 | 7 | 2 |
| *Glyma08g14270*  (unknown) | 1 | 2 | 0 | 1 | 1 | 0 | 0 | 0 | 0 | 0 | 0 | 0 | 7 | 0 |
| *Glyma08g12610*  (Armadillo) | 2 | 1 | 1 | 1 | 1 | 0 | 0 | 1 | 1 | 0 | 1 | 1 | 6 | 6 |
| *Glyma08g10480*  (C3HC zinc finger like) | 5 | 3 | 5 | 5 | 4 | 1 | 3 | 3 | 2 | 1 | 2 | 1 | 6 | 4 |
| *Glyma08g12210*  (putative serine esterase) | 1 | 5 | 2 | 2 | 2 | 0 | 1 | 1 | 1 | 1 | 1 | 0 | 6 | 2 |
| *Glyma08g13040*  (serine protein kinase) | 1 | 2 | 2 | 3 | 1 | 1 | 0 | 0 | 0 | 0 | 0 | 0 | 6 | 2 |
| *Glyma08g13150*  (Protein kinase domain) | 1 | 1 | 1 | 1 | 0 | 1 | 0 | 0 | 0 | 0 | 0 | 0 | 6 | 1 |
| *Glyma08g13880*  (Ricin type lectin) | 0 | 0 | 0 | 1 | 0 | 0 | 0 | 0 | 0 | 0 | 1 | 0 | 6 | 0 |
| *Glyma08g10760*  (mitochondrial beta-ketoacyl-ACP reductase) | 0 | 0 | 0 | 0 | 0 | 0 | 0 | 0 | 0 | 0 | 0 | 0 | 2 | 84 |

DAF: days after flowering
